# Oral RNAi in *Euschistus heros*: Evaluation of Gene Silencing Using Cationic Polymer-Stabilized dsRNA

**DOI:** 10.1007/s13744-026-01440-4

**Published:** 2026-09-16

**Authors:** Daniel Estiven Quiroga-Murcia, Danielle Ribeiro De Barros, Kristof De Schutter, Moises João Zotti

**Affiliations:** 1 Faculty of Agronomy “Eliseu Maciel”, Federal Univ of Pelotas, Capão Do Leão, 96160‑000 Brazil; 2 Faculty of Bioscience Engineering, Gent Univ, Ghent, Belgium

**Keywords:** RNA interference, *Euschistus heros*, Oral delivery, DsRNA stability, Gene silencing, Cationic polymers

## Abstract

*Euschistus heros*, a key pest of soybean in the Neotropical region, represents a growing challenge to sustainable agricultural production. With the urgent need for more targeted and environmentally safe control strategies, RNA interference (RNAi) emerges as an innovative tool. This study investigated the gene-silencing potential of RNAi targeting *V-ATPase* and *act*, two genes involved in essential cellular processes. The sensitivity of *E. heros* to RNAi in adult and immature stages was evaluated by microinjection of dsRNA, as well as the ex-vivo degradation of double-stranded RNA (dsRNA) by nucleases present in saliva and hemolymph. Additionally, an oral delivery strategy based on conjugation of dsRNA to a cationic polymer was studied. Microinjection of dsRNA resulted in high sensitivity, particularly in immature individuals, and induced strong gene-silencing effects associated with increased mortality. Intense nucleolytic activity was detected in saliva, underscoring the need for protective carriers for dsRNA. Oral exposure to conjugated dsRNA effectively protected the molecules from enzymatic degradation and, in addition, exhibited effects on insect survival and gene expression. These findings highlight the strong sensitivity of *E. heros* to dsRNA delivered by microinjection and underscore the potential of RNAi approaches for the development of tailor-made bio-insecticides, while emphasizing the need to refine delivery strategies to fully realize their practical applications in agricultural pest management.

**Supplementary Information:**

The online version contains supplementary material available at https://doi.org/10.1007/s13744-026-01440-4.

## Introduction

The brown soybean bug, *Euschistus heros* (Hemiptera: Pentatomidae), is a polyphagous insect with a wide host range, affecting important crops such as cotton, maize, and soybean (Malaguido and Panizzi 1999; Moraes et al. 2005; Sosa-Gómez and da Silva 2010; Panizzi et al. 2012, 2022). With a widespread distribution in Latin America and southern Brazil, Argentina, Paraguay, Uruguay, Bolivia, and Panama, this insect pest is considered one of the most important pests in South America (Sosa-Gómez et al. 2009; Saluso et al. 2011; Panizzi 2015). In the absence of these cultivated crops, *E. heros* can survive on weeds and trees, which act as reservoirs of the insect (Medeiros and Megier 2009; Costa et al. 2020; Panizzi et al. 2022). This insect, also called brown stinkbug, causes direct damage by feeding on pods and grains, depleting photo-assimilates and by injecting enzymes, such as amylases, which facilitate their feeding at the expense of grain quality and weight (Silva et al. 2012; Tessmer et al. 2022). Although various strategies—including biological control, cultural practices, and the use of resistant varieties—can be employed in the management of *E. heros*, chemical control remains the most widely used approach (Rossetto et al. 1986; Lourenção et al. 1987; Panizzi 2008; Michereff et al. 2019; Lucini et al. 2021; Panizzi et al. 2022). However, the intensive use of chemical control has a detrimental impact on the environment and human health and has led to the development of insecticide resistance in *E. heros* (IRAC n.d.; Sosa-Gómez et al. 2009; Sosa-Gómez and da Silva 2010).

Since the discovery of RNA interference (RNAi) in insects, researchers have focused on exploiting this naturally occurring mechanism for its potential in pest control. RNAi is initiated upon the detection of double-stranded RNAs (dsRNAs) in the cytosol. *Dicer* nucleases process the dsRNA into 18–24 bp small interfering RNAs (siRNAs) that are incorporated into the RNA-induced silencing complex (RISC). The siRNA guides the RISC to complementary RNA sequences that are cleaved by the Argonaute subunit of the RISC (Preall and Sontheimer 2005; Wilson and Doudna 2013). The presence of orthologs of RNAi-related genes in the *E. heros* transcriptome, encoding proteins involved in dsRNA and siRNA transport, RISC complex formation, and target mRNA degradation, suggests a functional RNAi machinery in this insect (Cagliari et al. 2020a). Sensitivity to RNAi was shown by microinjection of immature nymphs and adults with dsRNA targeting essential genes, resulting in high mortality, even at very low doses, and sublethal phenotypes such as low fecundity and developmental defects (Fishilevich et al. 2016; Castellanos et al. 2019). In addition, transgenerational effects or parental RNAi, in which adult females are treated with dsRNA and the phenotype is observed in the offspring, has been shown in this insect (Fishilevich et al. 2016; Castellanos et al. 2019; Cagliari et al. 2020b, 2021).

A promising target for a RNAi-based approach is the *V-ATPase* gene encoding the V-type vacuolar ATPase, an essential enzyme involved in proton transport across cell membranes and involved in acidification of intracellular vesicles, protein degradation, activation of hydrolases, pH homeostasis, and generation of proton motive force for ATP synthesis (Nishi and Forgac 2002; Forgac 2007). RNAi-mediated silencing of *V-ATPase* in insects leads to dehydration, significant weight reduction, impaired molting, structural damage to midgut cells, morphological alterations in the midgut, such as brush border membrane disruption, and thinning of the epidermal membrane (Liu et al. 2022; Zhang et al. 2023; Wang et al. 2024). The actin gene plays a crucial role in the formation of the cytoskeleton and is involved in essential cellular processes such as cell motility, muscle contraction, cell division, growth, as well as in cell signaling and morphology (Mitchison and Cramer 1996; Pruyne and Bretscher 2000; Baum and Perrimon 2001; Rosa et al. 2010). Its silencing at the cellular level can lead to morphological abnormalities (Rosa et al. 2010), as well as reductions in insect fecundity (Dong et al. 2024).

While RNAi is a promising strategy for pest control, its application in the field is hampered by several barriers. One of these challenges is the efficient delivery of the dsRNA molecules (Zotti et al. 2018; Silver et al. 2021; Palli 2023). In the case of *E. heros*, microinjection of ds*act* and ds*V-ATPase* leads to 95% mortality while oral exposure only results in 33% and 30% mortality, respectively (Castellanos et al. 2019). Similarly, in *Nezara viridula*, oral exposure to ds*V-ATPase* results in 43% mortality, whereas microinjection increases mortality to up to 90% (Sharma et al. 2021a). These findings underscore the variability in RNAi susceptibility depending on the delivery method. The reduced efficacy of RNAi following oral administration is attributed to the limited stability of dsRNA within the insect gut. In both *E. heros* and *N. viridula*, substantial dsRNA degradation activity has been observed in the saliva, primarily mediated by endonucleases that initiate dsRNA digestion within 10 min of exposure (Castellanos et al. 2019; Sharma et al. 2021a).

The objective of this research was to evaluate the potential of a cationic polymer to improve the susceptibility of adult and immature *E. heros* to ds*V-ATPase* administered through the oral route. Therefore, the enzymatic dsRNA degradation capacity of saliva and hemolymph was assessed. To overcome degradation of the dsRNA, the effect of complexation with a cationic polymer was evaluated.

## Materials and methods

### Insect rearing

*Euschistus heros* specimens came from a field-collected population maintained at the molecular entomology laboratories of the Federal University of Pelotas and Ghent University. Insects were reared in plastic containers under controlled conditions (24°C, 60% RH, and 16:8 h of light and dark) and fed fresh bean pods (P*haseolus vulgaris*), peanut seeds (*Arachis hypogaea*), and sunflower seeds (*Helianthus annuus*). Rearing containers and diet were renewed every 4 days.

### RNA extraction for the synthesis of dsRNA in E. heros

Total RNA was extracted from a single adult *E. heros* by homogenization in TRIZOL (Invitrogen) following the manufacturer’s instructions. RNA integrity was assessed by 1.5% agarose gel electrophoresis, and concentration was measured using a NanoVue Plus spectrophotometer (GE Healthcare). cDNA was synthesized with the GoScript Reverse Transcription System (Promega) using oligo(dT) and 5 µg of RNA.

The *V-ATPase* subunit A and the control gene *gfp* (Table 1) were amplified using specific primers. The *gfp* template was obtained from plasmid pIG1783F (Pöggler et al. 2003) grown in *Escherichia coli* DH5α and purified with the PureYield Plasmid Miniprep System (Promega). PCRs were prepared in 25 µL reactions using GoTaq® G2 Flexi DNA Polymerase (Promega), with the following cycling profile: 94 °C for 5 min; 30 cycles of 94 °C for 30 s, 55 °C for 45 s, and 72 °C for 55 s; and a final extension at 72 °C for 10 min. Amplicons were visualized in 1.5% agarose gels, purified with the Wizard® SV Gel and PCR Clean-Up System (Promega), and quantified by NanoVue.

**Table 1:** Primers used for sequencing, dsRNA synthesis, and RT-qPCR in *Euschistus heros*

	**Gene**	**Primer**	**Sequence (5′−3′)**	**Amplicon size (bp)**
Sequencing	*gfp (pIG1783f plasmid)*	*gfp*-F	TCGTGACCACCCTGACCTAC	560
		*gfp*-R	TCGTCCATGCCGAGAGTGAT	
	*V-ATPase subunit A E. heros*	Eh-V-ATP-F	GAGAGGGAGGGATCTGTTTCA	459
		Eh-V-ATP-R	GGCCAGGTCGTAGAAGGAAA	
dsRNA	*gfp (pIG1783f plasmid)*	ds*gfp*-F	TAATACGACTCACTATAGGGAGATCGTGACCACCCTGACCTAC	606
		ds*gfp*-R	TAATACGACTCACTATAGGGAGATCGTCCATGCCGAGAGTGAT	
	*V-ATPase subunit A E. heros*	dsEhV-ATP-F	TAATACGACTCACTATAGGGAGAGAGAGGGAGGGATCTGTTTCA	505
		dsEhV-ATP-R	TAATACGACTCACTATAGGGAGAGGCCAGGTCGTAGAAGGAAA	
	*act-2 (KT369806.1)*	dsEh-act-2-F	TAATACGACTCACTATAGGGAGAGATGACCCAGATCATGTTTGAGAC	508
		dsEh-act-2-R	TAATACGACTCACTATAGGGAGACAAGATTCCATACCCAAGAAGGAAG	
RT-qPCR	* 18 s ribosomal RNA*	RT18SrRNA-F	TACAACAAGACAACGCTCGC	150
		RT18SrRNA-R	TTGCGCTCAGTGACATCTCT	
	*Ribosomal protein L32e*	RT-rpl32-F	TCAGTTCTGAGGCGTGCAT	175
		RT-rpl32-R	TCCGCAAAGTCCTCGTTCA	
	*Dicer 2*	RT-DCR2-F	GAAGCAGGATAACCTCCTAA	156
		RT-DCR2-R	GGATGCAATTGTTCTACTGGA	
	*Ago 2*	RT-AGO2-F	GACCATCTCCACAACAAATG	113
		RT-AGO2-R	GTCAGAGGATTGAGGTCTAATA	
	*V-ATPase subunit A*	RT-EHV-ATP-F	CCCTTATTGACTGGTCAGCGT	100
		RT-EHV-ATP-R	TGACAGTTTTACCGCAACCG	
	*act-2*	RT-EHACT2-F	ATCACCAACTGGGACGACAT	100
		RT-EHACT2-R	GAGCCTCAGTCAGGAGGATG	

PCR products were cloned into pGEM-T Easy (Promega) and transformed into JM109-competent cells by heat shock. Positive colonies were identified by colony PCR, cultured in liquid medium supplemented with ampicillin, and the plasmids purified and sequenced bidirectionally (Supplementary information: Sequences 1–2). Plasmids were also digested with EcoRI (Promega) for additional confirmation.

The templates for dsRNA synthesis were obtained by PCR with specific primers that added the T7 promoter to the sequences in both directions (Table 1). For the microinjection of immatures, additionally, the *actin* gene (*act-2*; GenBank accession KT369806.1), previously described by Fishilevich et al. (2016) and referred to as *muscle actin* by Castellanos et al. (2019), was used (Supplementary information, Sequence 3). The amplification protocol for the dsRNA templates included 2.5 μL of 10X PCR buffer (Invitrogen), 1 μL of 10 mM dNTPs, 2.5 μL of 50 mM MgCl_2_ (Invitrogen), 2 μL of F primer at 10 mM (IDT), 2 μL of R primer at 10 mM (IDT), 2 μL of cDNA, 0.125 μL of recombinant Taq DNA Polymerase (5 U/μL) (Invitrogen), and 12.875 μL of water for a final volume of 25 μL. Amplification conditions were one denaturation cycle at 95 °C for 3 min, followed by 40 cycles of 30 s at 95 °C, 30 s at 60 °C, 1 min at 72 °C, and a final extension cycle at 72 °C for 10 min. The correct amplification and concentration of PCR products was verified by 1.5% agarose gel electrophoresis.

dsRNA was synthesized and purified using the MEGAscript™ RNAi Kit (Thermo Fisher), diluted in nuclease-free water, and stored at − 80°C. Product integrity and concentration were verified by agarose gel electrophoresis and NanoVue.

### Microinjection

For microinjections, insects were anesthetized with diethyl ether. Adults were immobilized on double-sided adhesive tape fixed to a slide, while immatures were placed on 1.5% agar in Petri dishes.

Adult insects were injected using a FemtoJet 4i (Eppendorf) equipped with glass capillary needles. Two experiments were performed: the first tested doses of 15, 28, and 33 ng/mg; the second tested 28, 33, and 40 ng/mg with three replicates per treatment and 10 insects per replicate. Dilutions of ds*V-ATPase* were prepared at concentrations of 450, 840, 990, and 1200 ng/μL. A volume of 2 μL was injected into the coxal corium of the right third leg, corresponding to doses of 900, 1680, 1980, and 2400 ng dsRNA per insect (equivalent to approximately 15, 28, 33, and 40 ng/mg body weight, respectively, based on an average adult weight of 60 mg). The same injection volume was used for control treatments, water and dsgfp (Cagliari et al. 2020a). Immatures (third instar) were injected using a Nanoject III (Drummond Scientific). Due to high mortality in second instars (Villas Bôas and Panizzi 1980), only third instars (≈ 5 mg) were used. Insects received 0.2 μL of ds*V-ATPase* or ds*act* at 700 ng/μL (28 ng/mg), with water and ds*gfp* as controls. Three replicates per treatment were performed, each with 10 insects.

After injection, insects were maintained under rearing conditions, and mortality and behavioral changes were recorded daily.

### Ex vivo degradation of dsRNA and dsRNA polyplexed

The ability of saliva, hemolymph, and midgut extracts to degrade dsRNA was evaluated.

For saliva collection, adult insects were placed in a Petri dish at −  20 °C and subsequently fixed on a dish using double-sided adhesive tape. When insects began to recover, saliva secretion from the rostrum was stimulated (Peiffer and Felton 2014) and collected using a glass capillary tube, transferred to an Eppendorf tube kept on ice, centrifuged periodically, and stored at − 80°C.

Hemolymph was obtained using a similar procedure. Insects were anesthetized at −  20 °C and fixed on adhesive tape. The rostrum was isolated from the body to prevent contamination with saliva. An incision was made in the coxal corium and hemolymph was collected with a capillary tube into an Eppendorf microtube containing phenylthiourea (PTU) (Christiaens et al. 2018), kept on ice, centrifuged periodically, and stored at − 80°C.

Ex vivo degradation assays were performed using both naked dsRNA (ds*V-ATPase* and ds*gfp*) and dsRNA polyplexes at a concentration of 200 ng dsRNA/µL. For each assay, 30 µL of sample were incubated with either 3 µL of saliva or 3 µL of hemolymph, separately, in 150 µL microtubes.

The cationic polymer used for polyplex formation, poly(2-aminoethylmethacrylate) (pAEMA), was synthesized from aminoethylmethacrylate (AEMA) monomers following a modified protocol from Christiaens et al. (2018). The pAEMA H formulation used in the present study has been previously characterized in greater detail by Vereecken et al. (2026), including particle size, zeta potential, and stability under different pH, temperature, and ionic strength conditions. Briefly, 1 g of AEMA was dissolved in double-distilled water acidified to pH 4 with 0.5 M HCl and 11 mg ammonium persulfate (APS) initiator was added. The solution was degassed for 30 min under argon, and free radical polymerization was initiated by heating at 70 °C with stirring for 24 h. The resulting polymer was purified by dialysis against acidified double-distilled water (pH 4) using 3500 Da molecular weight cutoff membranes (SpectraPor) for 24 h, followed by lyophilization. Polyplexes were prepared following Christiaens et al. (2018) at a 2:1 polymer-to-dsRNA ratio, using stock solutions of 2000 ng/µL polymer and 2500 ng/µL dsRNA. Formation of dsRNA–pAEMA complexes were confirmed by agarose gel electrophoresis (Figure S1, Supplementary Information).

For degradation assays, samples were incubated at 25 °C and 5 µL aliquots were collected at 1, 10, 30, 60, 120, and 180 min. To evaluate intrinsic stability, 30 µL of naked or polyplexed dsRNA was mixed with 3 µL of water and incubated at 25 °C, with aliquots collected at 10, 30, 60, 120, and 180 min. For decomplexation, each aliquot was immediately mixed with 7.5 µL of 1% SDS, vortexed, and stored on ice prior to electrophoresis on 1.5% agarose gels. Additionally, an aliquot of each dsRNA–polymer complex was incubated in saliva for 72 h and analyzed by electrophoresis after SDS treatment.

Band intensity was quantified using GelAnalyzer 23.1.1. Bands from each treatment were selected, and intensity profiles were obtained for each time point.

### Oral exposure

As a vehicle for dsRNAs, a modified version of the “D3” diet (Hayashida et al. 2018) was used. The amount of water was doubled to facilitate dsRNA dilution and obtain a semi-solid diet. The diet consisted of 13% peanuts, 2% sucrose, 11.1% soybeans, and 74% nuclease-free water; solid components were ground prior to water addition. dsRNAs were incorporated into the nuclease-free water at a concentration of 500 ng/mg. The treatments included ds*V-ATPase*, ds*act*, ds*V-ATPase* polyplexes, ds*act* polyplexes, and controls (water, ds*gfp*, and ds*gfp* polyplexes), all at the same concentration. Polymerization was performed as previously described. To assess the polymer effect alone, an additional treatment consisted of diet supplemented only with polymer, with three replicates of 10 insects.

Diet palatability was evaluated by estimating consumption over two consecutive 24-h exposure periods. Consumption was calculated from the weight difference of the diet containers, with an average intake of 4.2 mg per insect. The lowest consumption was observed in the dsV-ATPase polyplex treatment (3.17 mg per insect), whereas diet supplemented with dsgfp showed the highest intake (5.95 mg per insect) (Figure S4).

For bioassays, 40 mg of diet were placed into feeding chambers made from 150-µL microtube caps and covered with a thin Parafilm layer to minimize evaporation. Diet was replaced after 24 h, and removed after 48 h, at which point consumption was recorded and 2-cm^2^ bean pieces were provided. Each treatment consisted of three replicates of 10 third-instar insects. Mortality was recorded for up to 7 days after diet removal.

### Evaluation of gene expression

Gene expression in *E. heros* adults was evaluated after microinjection. Fifteen insects were injected per treatment. For each sampling time, three insects were collected, immediately frozen in liquid nitrogen and stored at −  80 °C until RNA extraction. Total RNA was extracted using TRIZOL (Invitrogen) following the manufacturer’s instructions, dividing each sample to obtain ≤ 100 mg of tissue. Homogenization was carried out manually with pestles in 2-mL tubes.

The expression of V-ATPase and act genes, as well as the activation of the RNAi machinery, was assessed following oral exposure to target or control dsRNA. For each treatment, three biological replicates consisting of 10 insects were established. From each replicate, three insects were collected at 24 and 96 h after removal of the second container with treated diet. RNA was subsequently extracted using the RNeasy Plus kit (Qiagen).

RNA was quantified by spectrophotometry, and quality was verified by 1.5% agarose gel electrophoresis. cDNA was synthesized from 1000 ng of total RNA using the iScript cDNA Synthesis Kit (BioRad), or the SuperScript™ IV kit (Thermo Fisher) for oral exposure experiments.

RT-qPCR was performed on a CFX 96 Real-Time System (BioRad). Primer efficiency was determined by standard curves, and specificity by melting curves (60–90°C). Reactions consisted of 20 µL containing: 10 µL of BrightGreen 2 × MasterMix (abm) or GoTaq® MasterMix (Promega), 0.5 µL of each primer (10 mM), 1 µL cDNA, and water. Cycling conditions were 95 °C for 3 min, followed by 40 cycles of 95 °C for 15 s, 60 °C for 1 min, and 72 °C for 20 s. Melting curves were run from 60–95°C. All reactions were performed in 96-well plates with three technical replicates.

Gene expression was calculated using the 2^−ΔΔCt^ (Livak and Schmittgen 2001) method in RStudio. *RPL32* and *RNA18S* were used as reference genes (Cagliari et al. 2020a).

For *Ago2* and *Dicer*2, the reference group was the water-injected treatment. In oral exposure the reference was the diet mixed with water. For *V-ATPase* and *act*, the reference was the treatment with ds*gfp*, either microinjected, or added to diet depending on the exposure method.

### Statistical analysis of the data

Mortality data were analyzed using Kaplan–Meier survival curves followed by log-rank tests to compare survival among treatments. For multiple pairwise comparisons, *p*-values were adjusted using the Holm-Sidak procedure.

Diet consumption data were analyzed using one-way analysis of variance (ANOVA) to assess differences among treatments. Prior to analysis, data were evaluated for normality and homogeneity of variances. Homoscedasticity was assessed using Brown–Forsythe and Bartlett’s tests. Statistical significance was set at *p* < 0.05.

Gene expression data were first tested for normality using the Shapiro–Wilk test. Non-normal data were transformed using a Box-Cox transformation with the transformTukey() function from the rcompanion package in R, which identifies the optimal lambda (power transformation) to stabilize variance and approach normality. ANOVA was then applied to transformed data to test for differences among treatments.

Mortality and gene expression statistical analyses were conducted in R version 2026.01.2 + 418, using packages rcompanion (for Box-Cox transformation), survival (Kaplan–Meier and Log-Rank test), and multcomp (Tukey’s test). Diet consumption analyses were performed using GraphPad Prism software.

## Results

### Euschistus heros sensitivity to RNAi

**Fig. 1:**
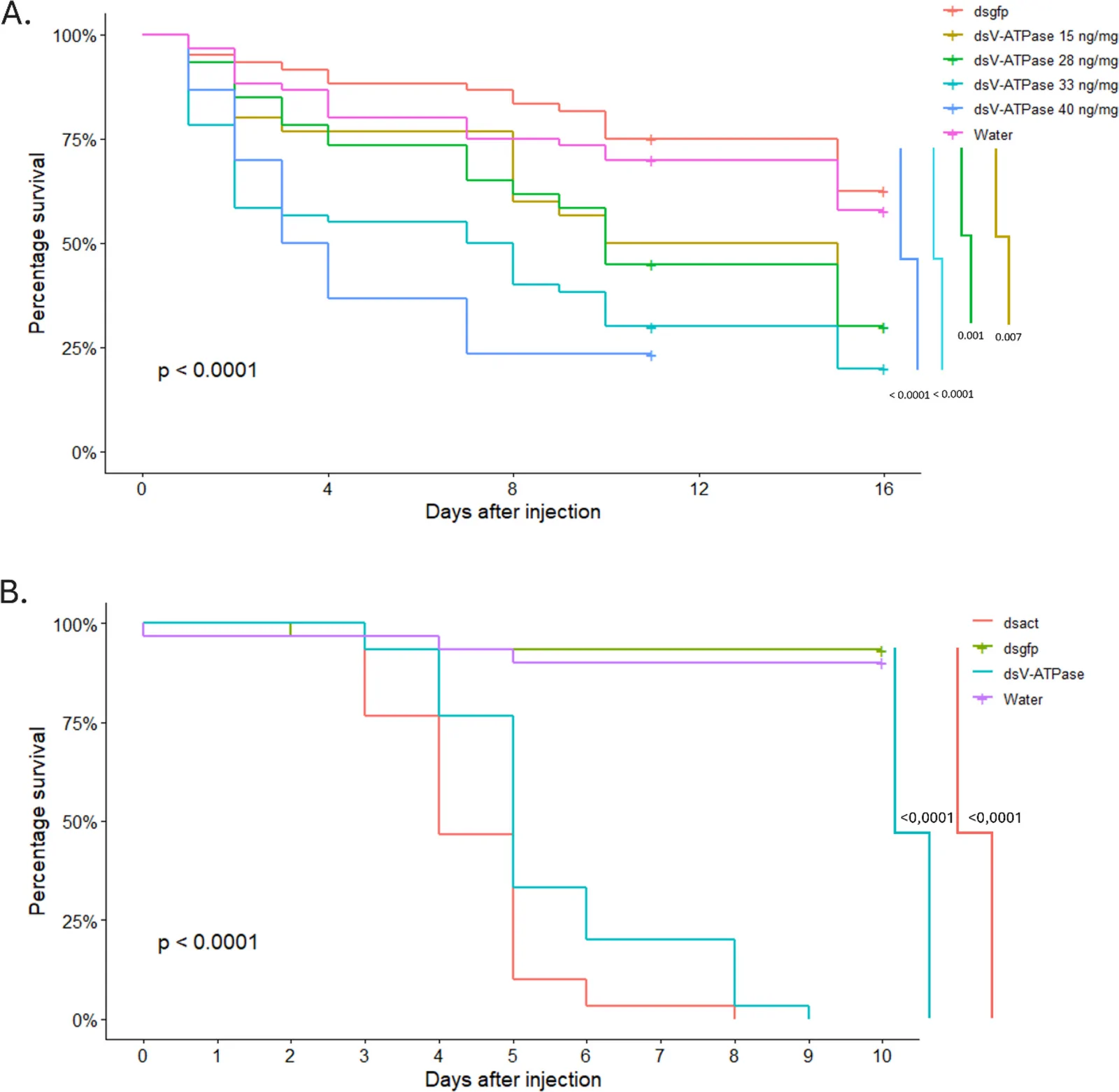
Kaplan–Meier survival curves after microinjection of dsRNA. **A** Combined survival analysis of *Euschistus heros* adults injected with four doses of ds*V-ATPase* (15, 28, 33, and 40 ng/mg insect). Controls included dsgfp (28 ng/mg insect) and water. Two independent experiments were performed: the first tested doses of 15, 28, and 33 ng/mg, with survival monitored up to 16 days; the second tested doses of 28, 33, and 40 ng/mg, with survival monitored up to 11 days. Survival data for the shared doses (28 and 33 ng/mg) were combined for Kaplan–Meier analysis. The 40 ng/mg treatment is therefore shown up to day 11, corresponding to the duration of the second experiment. Statistical differences are indicated by bars on the graph, with *p*-values adjusted using the Holm-Sidak method. **B** Survival curve of *Euschistus heros* third-instar insects microinjected with ds*V-ATPase* and ds*act* (28 ng/mg insect). Controls included dsgfp (28 ng/mg insect) and water. Mortality was monitored for 10 days. Statistical differences are indicated by bars on the graph, with *p*-values adjusted using the Holm-Sidak method

Microinjection of dsRNA targeting the *V-ATPase* gene at all concentrations tested had a significant (*p* < 0.001) impact on the survival of *E. heros* adults and nymphs when compared to the control treatments ds*gfp* and water (Fig. 1A). In addition, a dose-dependent response was observed, with higher dsRNA concentrations resulting in increased mortality rates (Fig. 1A). In immature insects, treatment with dsRNA targeting the *act* gene resulted in significant effects on the survival of individuals (*p* < 0.001) (Fig. 1A).

In adults, a gradual increase in mortality was observed from the second day post-microinjection, with the highest dose of ds*V-ATPase* (40 ng/mg) inducing an accelerated mortality progression, exceeding 75% by day 7 (Fig. 1A). The overall mortality trajectory suggested a non-linear progression over time, with an initial phase of increased mortality followed by a second phase of sustained decline across all doses. In immature insects, an abrupt decrease in survival was recorded after the third day. Mortality progression for the ds*act* treatment was faster than for the ds*V-ATPase* treatment, although by the end of the experiment survival in both treatments had dropped below 5% (Fig. 1B).

**Fig. 2:**
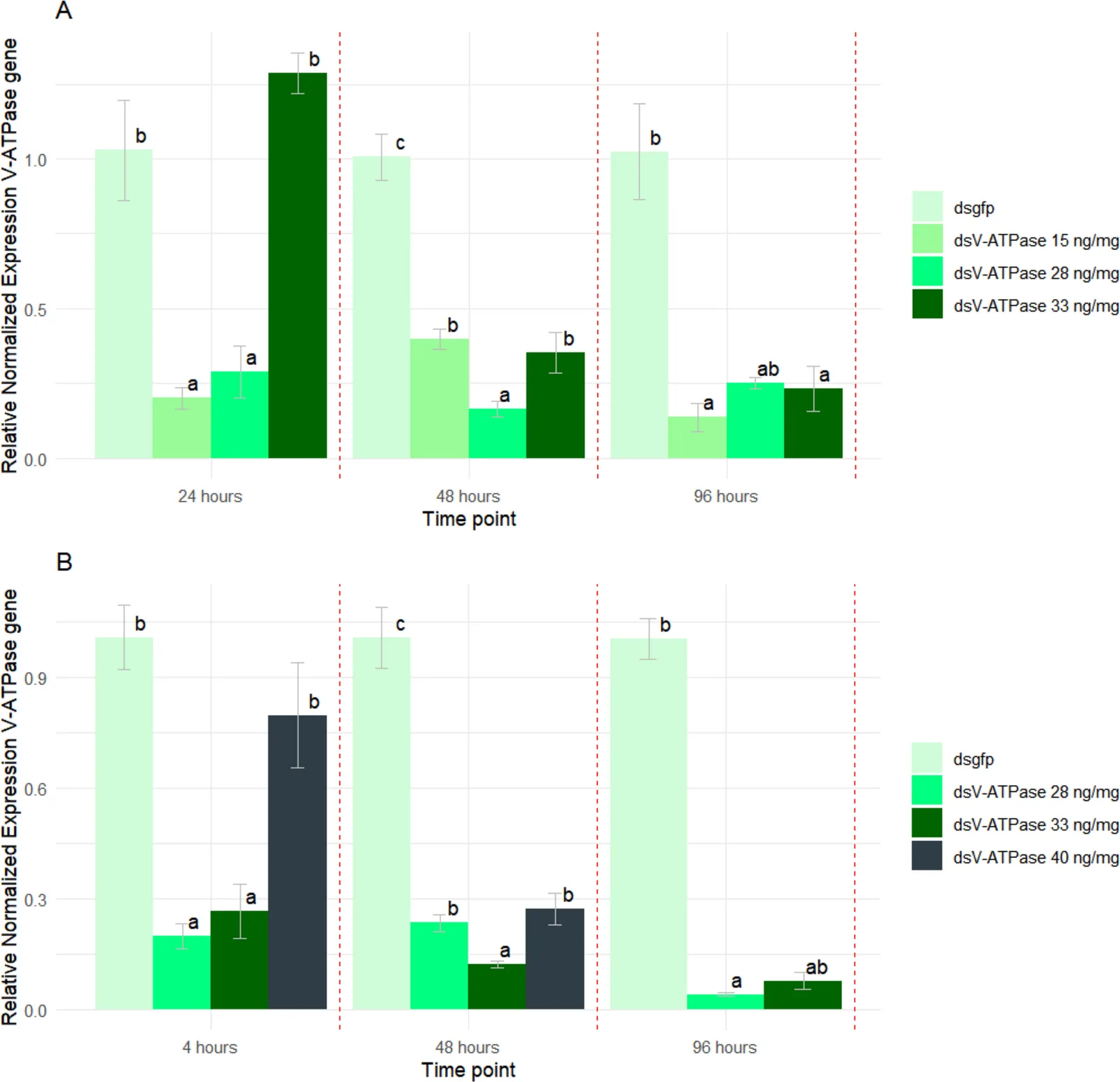
Normalized relative expression of the *V-ATPase* gene in adults *Euschistus heros* microinjected with ds*V-ATPase*. **A** Experiment 1: Insects were microinjected with ds*V-ATPase* at concentrations of 15, 28, and 33 ng/mg insect, with evaluations performed at 24, 48, and 96 h. **B** Experiment 2: Insects were microinjected with ds*V-ATPase* at concentrations of 28, 33, and 40 ng/mg insect, with evaluations performed at 4, 48, and 96 h. In both experiments, controls included ds*gfp* (28 ng/mg insect) and water. The normalized relative expression was calculated using the 2^−ΔΔCT^ method, employing two reference genes, *RPL32* and *18SR*, and further normalized to the control treatment ds*gfp*. Letters above the bars indicate statistical differences between treatments as determined by Tukey’s test (*p* = 0.05)

Microinjection of dsRNA in adults resulted in silencing of the *V-ATPase* gene at all doses tested, showing significant differences (*p*-value < 0.001) in transcript levels between individuals treated with ds*V-ATPase* and those treated with the ds*gfp* control (Fig. 2). The effects on gene expression were detectable as early as 4 h post-microinjection and became more pronounced over time, reaching a reduction in transcript levels between 77 and 95% at 96 h. Interestingly, in the insects treated with the highest dose of ds*V-ATPase* no silencing was observed during the first 4 to 24 h (Fig. 2).

**Fig. 3:**
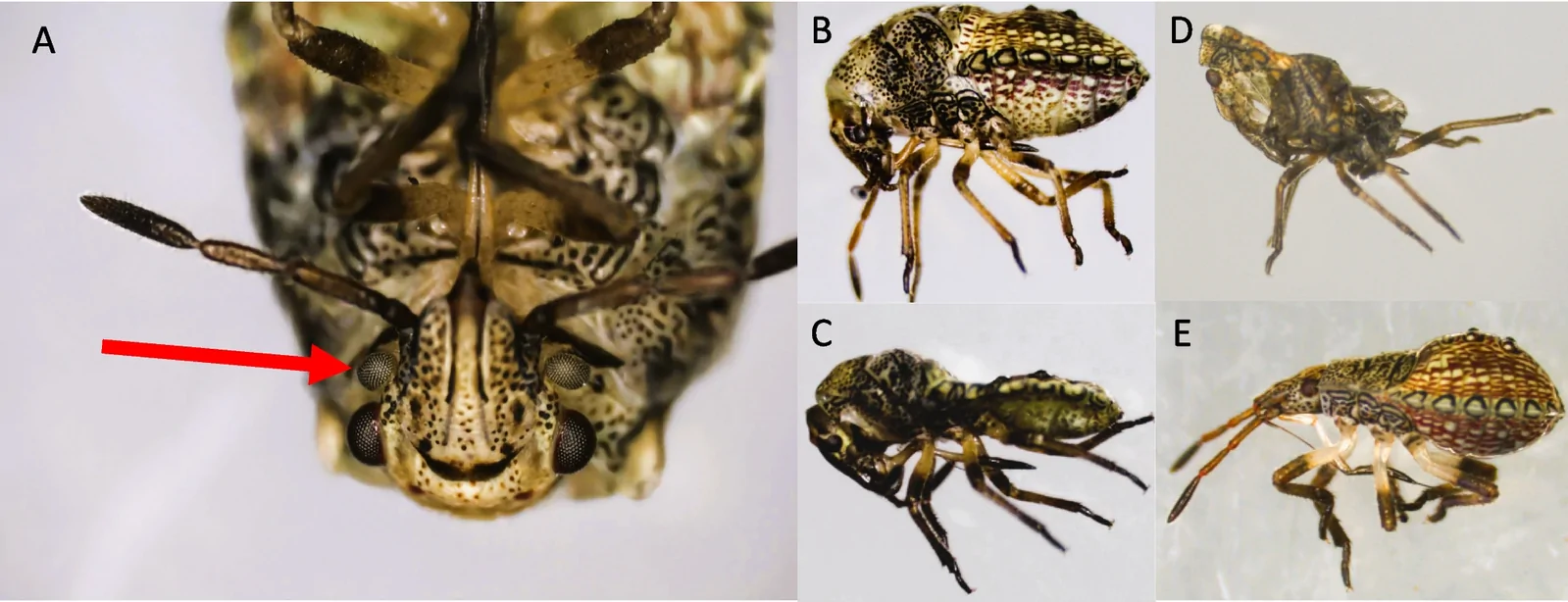
Phenotypes observed in immature *Euschistus heros* four days after microinjection with ds*V-ATPase* (28 ng/mg). **A** Retention of the old cuticle (arrow); **B** initial morphological alterations, including anteroposterior compression and swelling; **C** hemolymph leakage following cuticle rupture; **D** desiccation resulting from hemolymph loss and anteroposterior compression; **E** dead insect from the control group, showing the characteristic morphology

From the third day after microinjection in immature individuals, several phenotypes were identified that led to the death of the insects. Among the most frequent phenotypes was an inability to molt, which led to the insects becoming trapped in the cuticle of the previous instar, altering the body shape and finally causing death. Impaired coordination of movement, lethargy, swelling, and, in some cases, hemolymph leakage due to cuticle rupture were also observed. In addition, the lack of feeding was noted in some individuals, who at death showed marked dorsoventral compression (Fig. 3). In adults, no anatomical changes were detected following ds*V-ATPase* injection; however, lethargy and irregular movements were observed.

### Ex vivo degradation of naked dsRNA and dsRNA polyplexes

**Fig. 4:**
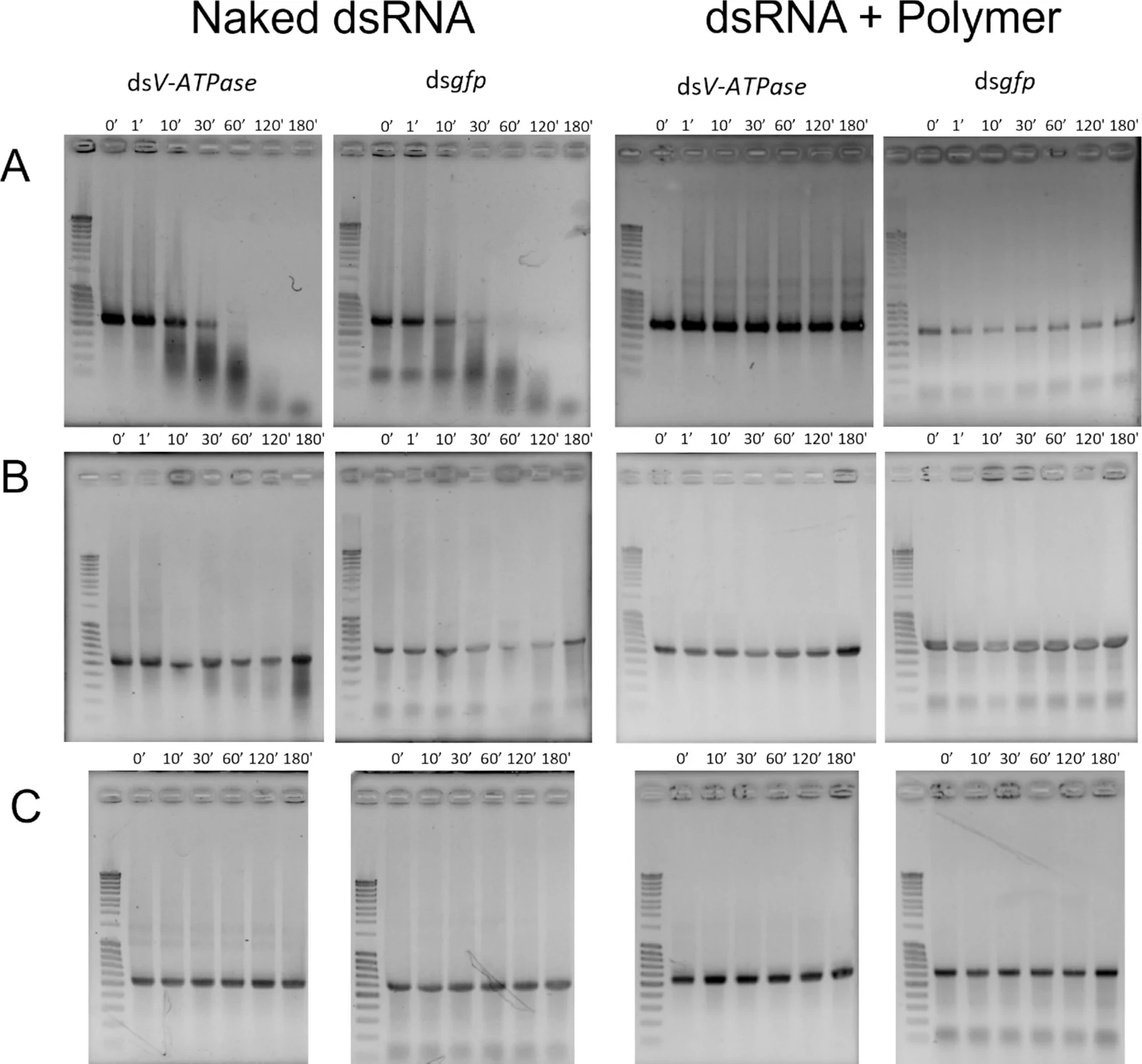
Ex vivo degradation of naked dsRNA and dsRNA polyplex in saliva and hemolymph from *Euschistus heros*. **A** Degradation of 30 µL of naked and polyplex ds*V-ATPase* and ds*gfp* (200 ng/µL) after incubation with 3 µL of *Euschistus heros* saliva. Samples were collected at 1, 10, 30, 60, 120, and 180 min at 25°C. **B** Degradation of 30 µL of naked and polyplex ds*V-ATPase* and ds*gfp* (200 ng/µL) following incubation with 3 µL of *Euschistus heros* hemolymph. Samples were collected at 1, 10, 30, 60, 120, and 180 min at 25°C. (**C**) Control degradation assay using 30 µL of naked and polyplex ds*V-ATPase* and ds*gfp* (200 ng/µL) incubated with 3 µL of nuclease-free water. Samples were collected at 10, 30, 60, 120, and 180 min at 25°C

The stability of dsRNA is crucial to induce an efficient RNAi response. When dsRNAs were mixed with saliva extracted from *E. heros*, significant degradation was observed after 30 min with only a faint band of dsRNA remaining after 60 min (Fig. 4). After 2 h, only low molecular weight fragments, a consequence of degradation, were visible on the agarose gel (Fig. 4). The protection of the dsRNA against degradation by the formation of polyplexes with the cationic polymer was clearly appreciated. Most of the dsRNA could be recovered from the polyplex after exposure to saliva (Fig. 4). Even after 72 h of incubation of the polymer-conjugated dsRNA in saliva, it was observed that the molecules retained their stability (Figure S2, Supplementary information).

In hemolymph, no degradation was observed after 180 min, although faint bands were observed in the electrophoresis of ds*gfp* (Fig. 4). At the end of the experiment, a band with a concentration similar to the initial level was still visible. Also, for the polyplexes, generated by mixing the dsRNA with the polymer, all the dsRNA in the experiment was recovered, with strong bands observed throughout all sampling times (Fig. 4).

The dsRNA exhibited high stability in RNase-free water, being resistant to temperature and the light incidence during incubation up to 180 min. In this case, both the naked and polymer-conjugated molecules were fully recovered, exhibiting an intense banding pattern on electrophoresis (Fig. 4).

### Oral exposure to dsRNA

**Fig. 5:**
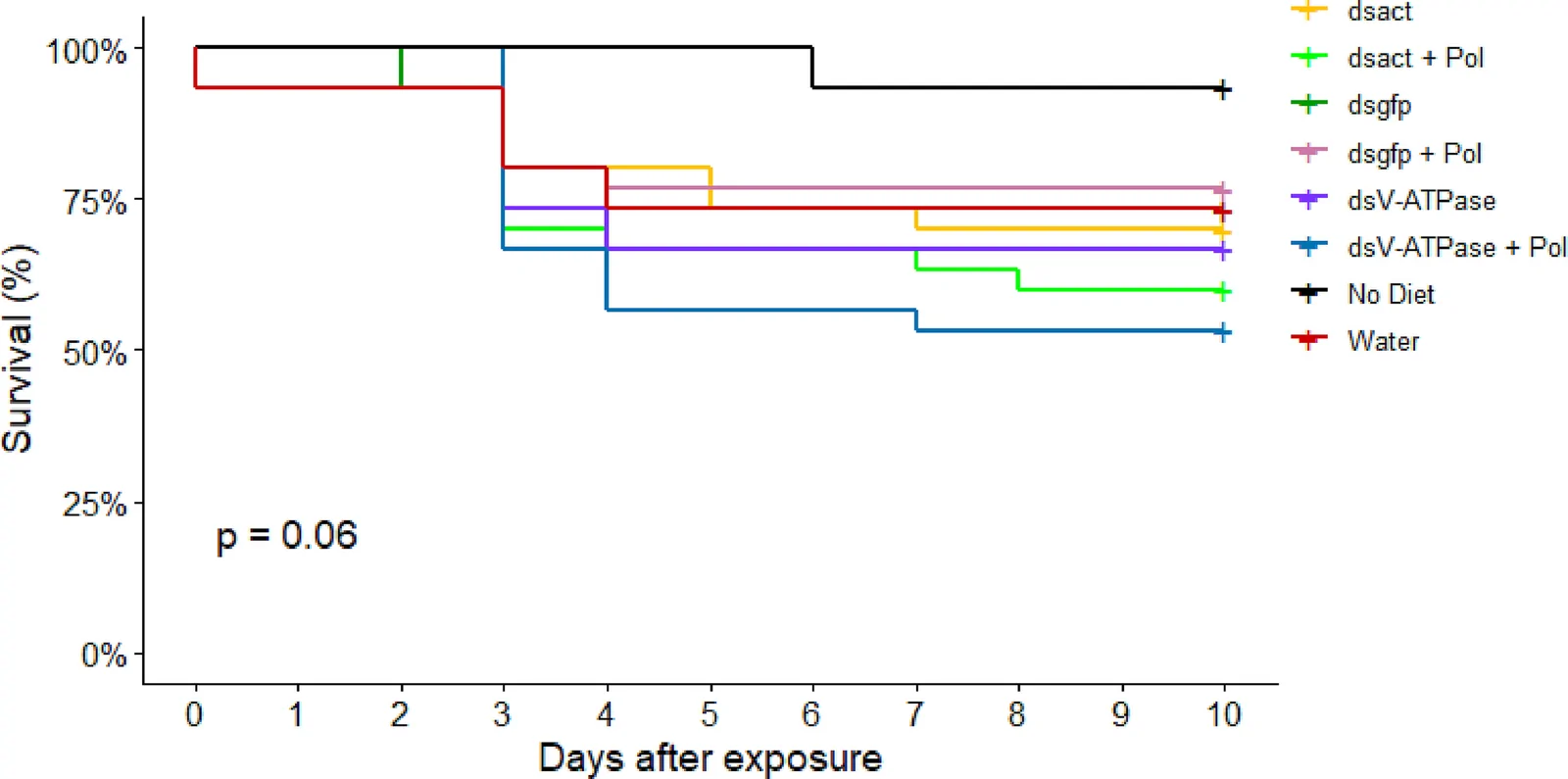
Kaplan–Meier survival curve of *Euschistus heros* third instar nymphs fed an artificial diet containing naked and polyplex ds*V-ATPase* and ds*act*. Controls included beans or an artificial diet supplemented with naked and polyplex ds*gfp* or water. Mortality was monitored over 10 days. *p*-value < 0.05 was considered significant

*Euschistus heros* third instar insects were fed a semi-liquid diet supplemented with naked dsRNA, dsRNA polyplexes or control treatment. Diet consumption did not differ significantly among treatments (one-way ANOVA, *F* = 2.351, *p* = 0.0679), and variance homogeneity was confirmed (Brown–Forsythe and Bartlett tests, *p* > 0.05) (Supplementary information figure S4). Mortality ranged from 20 to 56% among treatments. The highest mortality was observed in insects fed the unconjugated polymer (56%), followed by ds*V-ATPase* polyplexes (47%) and ds*act* polyplexes (40%) (Fig. 5). Mortality in the control treatments and in insects fed naked dsRNA remained below 35%.

Kaplan–Meier survival analysis revealed no significant differences among the diet-based treatments (dsRNA naked, polyplexes, polymer alone, and diet-fed controls; *p* = 0.06). However, pairwise comparisons indicated significantly higher survival in the bean-fed group compared with insects treated with ds*V-ATPase* and ds*act* polyplexes.

To analyze the impact on gene expression following dsRNA ingestion, third instar larvae of *E. heros* were fed a semi-liquid diet containing free dsRNA and dsRNA conjugated with a cationic polymer. Insects were collected on the third and sixth day after the start of the experiment.

**Fig. 6:**
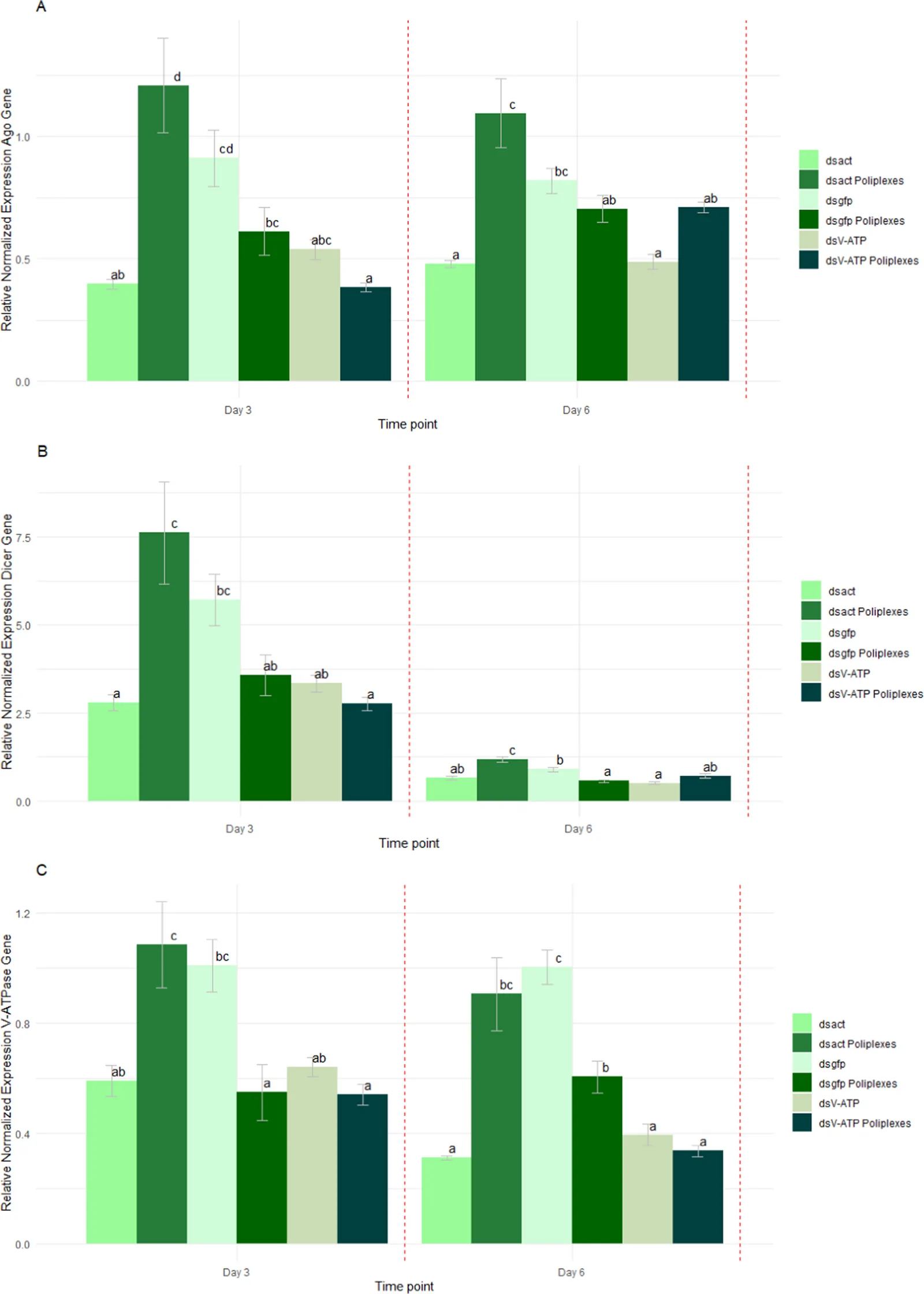

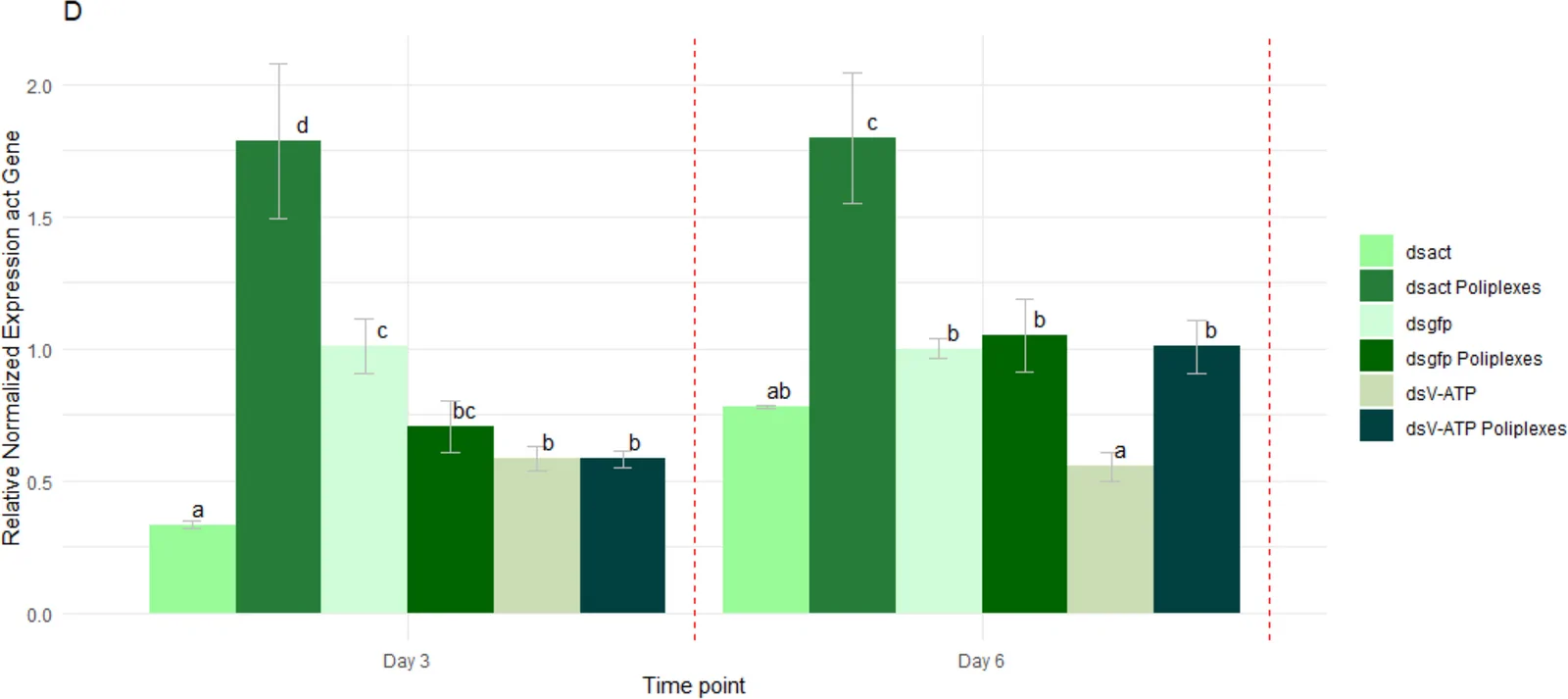
The normalized relative expression in third instar *Euschistus heros* fed an artificial diet mixed with a solution containing naked and polyplexes ds*V-ATPase*, and ds*act* (500 ng/mg diet). **A** *Ago* gene and **B** *Dicer* gene, which are components of the RNAi machinery, and the target genes, **C** *V-ATPase* and **D** *act*. The controls used were ds*gfp*, ds*gfp* polyplexed and water. Evaluations were performed at 3 and 6 days. In both experiments, normalized relative expression was calculated using the 2^-ΔΔCT method, using two reference genes, *RPL32* and *18SR*. Letters above the bars indicate statistical differences between treatments at the same time point, as determined by Tukey’s test (*p* = 0.05)

Insects that consumed the ds*act* diet exhibited a decrease in *act* gene expression on day three, although this expression increased again by day six, remaining 20% below the expression observed in the ds*gfp* treatment (Fig. 6). On the other hand, insects that ingested the diet with ds*act* and the polymer showed an increase in *act* gene expression at both sampling periods, reaching 80% more compared to the control diet with ds*gfp*.

A decrease in *V-ATPase* gene expression of 38–60% was recorded in insects feeding on the ds*V-ATPase* diet and the ds*V-ATPase* polyplexes diet compared to the ds*gfp* diet control, with these differences being statistically significant. However, this reduction was also observed in control treatment with the ds*gfp* plus polymer diet (Fig. 6).

*Dicer* gene expression was higher compared to the water diet control on day 3 sampling for all treatments, with highly significant differences. However, expression levels decreased by day 6 sampling, showing no significant difference compared to the same control.

The expression pattern of the *Ago* gene varied between samples. Low expression was detected in the treatment with the ds*act* diet in both samplings and with ds*V-ATPase* plus polymer in the day three sampling. The highest expression was observed in the ds*act* plus polymer treatment (Fig. 6).

## Discussion

*V-ATPase* is an essential enzyme involved in a plethora of cellular processes. In *E. heros*, RNAi-mediated silencing of the gene encoding the subunit A of *V-ATPase*, by microinjection of dsRNA targeting this gene, resulted in high adult mortality for all doses evaluated. The sensitivity of *E. heros* to dsRNA is remarkable, even at doses 40% lower than those evaluated before (Castellanos et al. 2019; Cagliari et al. 2020a). This is consistent with the high susceptibility of this insect to RNAi reported previously (Fishilevich et al. 2016; Castellanos et al. 2019; Cagliari et al. 2020a). Microinjection of dsRNA targeting *ATPase*, *N2B*, and *PP1* genes in *Halyomorpha halys* adults resulted in 100% mortality at 7 days, indicating higher susceptibility at this stage compared to *E. heros* (Mogilicherla et al. 2018).

In adults of *E. heros*, the non-linear mortality observed after microinjection may reflect a heterogeneous timing in the RNAi silencing of *V-ATPase*. Even under the same experimental conditions, differences in the timing and effectiveness of gene knockdown may cause some individuals to reach a critical functional level needed for survival sooner than others silencing (Rodrigues and Figueira 2016), leading to early death in the more sensitive subjects and delayed effects in the others. Further studies are required to elucidate the underlying mechanisms and validate these observations. In immatures, a single mortality peak was observed between days 4 and 5, consistent with findings in *Apolygus lucorum* following ds*V-ATPase* subunit A microinjection (Liu et al. 2019).

Mortality demonstrated a dose-dependent response. Although mortality rates became more similar across treatments from day 9 onwards, higher doses induced accelerated mortality. This may be advantageous for pest management, as it could reduce feeding damage and limit population growth more effectively. Therefore, the selection of an optimal dsRNA dose should balance the benefits of faster mortality against practical considerations, including dsRNA production costs, the application rates required under field conditions, and potential non-target or environmental effects associated with higher doses. *V-ATPase* gene expression was significantly affected by microinjection of ds*V-ATPase*, with even a strong silencing effect observed at an early stage (4 h post-injection).

Low dsRNA doses induced an early reduction in transcript levels, and this silencing effect was maintained or further enhanced throughout the experiment. In contrast, higher dsRNA doses exhibited a more complex temporal response, characterized by an initial increase in transcript levels at 4 h post-injection, followed by a progressive reduction at 48 and 96 h, when significant gene silencing became evident. These results suggest a dose-dependent dynamic response to dsRNA exposure, potentially reflecting an early stress or compensatory regulatory mechanism at higher dsRNA concentrations prior to the establishment of RNAi-mediated gene silencing. These findings are consistent with previous reports describing temporal variability in RNAi responses in insects (Castellanos et al. 2019). However, they differ from the observations by Cagliari et al. (2020a, b), who reported a recovery of gene expression 48 h after treatment. In the present study, silencing was generally sustained up to 96 h in most treatments compared to the dsgfp control. Similarly, in *Nezara viridula*, V-ATPase transcript levels were significantly reduced between 48 and 72 h following ds*V-ATPase* microinjection (Sharma et al. 2021a).

The RNAi machinery responds to microinjection with dsRNA by showing elevated expression levels that vary and do not follow a consistent pattern that would infer a specific alteration due to the microinjection. At higher doses, elevated *Ago* and *Dicer* expression values are observed, which could be related to the large number of dsRNA present. It is crucial to assess which dose is high enough to induce effective silencing without saturating the RNAi machinery, which could interfere with the silencing process.

In third instar insects, mortality was faster, even though doses were adjusted to insect weight, indicating that age is an important factor in susceptibility to RNAi. Previous studies in second instar insects exposed to ds*V-ATPase* showed 80% mortality on day eight, remaining at that level until day fourteen (Castellanos et al. 2019). In the present study, mortality on day eight reached 95% and increased to 100% on day nine.

For third instar insects treated with ds*act*, mortality reached 100% at day eight. In previous studies with second instar immatures, mortality was 60% at day seven with the same molecule (Castellanos et al. 2019), although Fishilevich et al. (2016) reported 100% mortality in second instar insects injected with ds*act*. This suggests that other factors, such as nutritional status and abiotic stress, may influence susceptibility to RNAi. In *N. viridula*, second instar immatures treated with ds*V-ATPase* reached 80% mortality by day seven, with mortality progression like that of *E. heros*, where after day three mortality occurs readily ((Sharma et al. 2021a)).

The silencing of *V-ATPase* gene in *E. heros* immatures resulted in phenotypes consistent with those reported in other insects, including lethargy, dehydration, and failure to molt to the next instar. The observed lethargy aligns with previous studies (Cagliari et al. 2020a). In *Hyphantria cunea*, silencing of the *V-ATPase* subunit A led to downregulation of chitinase (CHT) and insect cuticular protein genes (Zhang et al. 2023), highlighting the essential role of *V-ATPase* subunit A in cuticle metabolism and its influence on molting.

In this study, insects appeared confined within their old exoskeletons during the instar transition, suggesting that V-ATPase silencing may disrupt processes essential for successful ecdysis. This interpretation is further supported by the delayed onset of adult mortality, indicating that the effects of gene silencing are most pronounced during developmental stages preceding the attainment of full growth. The observed phenotype may reflect a physiological effect associated with V-ATPase knockdown, given its essential role in multiple cellular and energetic processes (Nishi and Forgac 2002; Forgac 2007). Future studies should investigate the potential effects of V-ATPase silencing on sexual development, as this stage may reveal transgenerational consequences, similar to those observed following the silencing of chromatin-remodeling ATPases (Fishilevich et al. 2016).

RNases are among the main barriers to RNAi, and their abundance varies considerably across insect tissues. For instance, in *Cylas puncticollis*, at least two midgut nucleases actively degrade dsRNA (Prentice et al. 2019). In *N. viridula* and *E. heros*, an endonuclease capable of dsRNA degradation has also been identified (Cagliari et al. 2020a; Sharma et al. 2021b). Our study confirms that saliva displayed a strong ability to degrade dsRNA and constitute a primary barrier for RNAi. Previous studies reported degradation in *E. heros* saliva, occurring within 10 min (Castellanos et al. 2019), while *N. viridula* saliva caused pronounced degradation after one hour (Sharma et al. 2021a).

In *Acyrthosiphon pisum*, the degradative capacity of saliva was assessed indirectly by observing the stability of dsRNAs in the diet after insect feeding, showing a remarkable degradation at 8 h compared to the insect control (Christiaens et al. 2014). *Lygus lineolaris* saliva took about 30 min to degrade dsRNA, whereas in hemolymph it did not show robust degradation capacity (Allen and Walker 2012).

The stability of both naked and polymer-conjugated dsRNA in *E. heros* hemolymph contrasts with previous studies (Cagliari et al. 2020a). But are consistent with the no degradation observed in *N. viridula* hemolymph (Sharma et al. 2021a). On the other hand, in *A. pisum*, dsRNA degradation was observed 3 h after the start of the experiment (Christiaens et al. 2014).

The low nuclease activity in hemolymph is consistent with the potent silencing effect by microinjection in *E. heros*, a phenomenon also observed in *Locusta migratoria*, where no dsRNA degradation is observed upon mixing with hemolymph, and this insect shows high sensitivity to RNAi by microinjection (Song et al. 2017). In the opposite case, *Manduca sexta* can degrade dsRNA in hemolymph, being more noticeable in the lepidopteran (Garbutt et al. 2013). The effect of nucleases in midgut tissues and contents has also been observed in *Bombyx mori* (Arimatsu et al. 2007), *L. migratoria* (Song et al. 2019), *Leptinotarsa decemlineata* and *Schistocerca gregaria* (Spit et al. 2017).

In *E. heros*, conjugation of dsRNA with a polymer provided broad protection against nuclease‐mediated degradation, similar to the effect observed when dsRNA was incubated with lipofectamine (Castellanos et al. 2019). Vereecken et al. (2026) previously characterized the physicochemical properties of polyplexes prepared with the same pAEMA H formulation. In that study, the polyplexes exhibited particle diameters below 100 nm and a positive zeta potential at N/P ratios ≥ 1, and remained stable under a range of pH, temperature, and ionic strength conditions. These physicochemical characteristics may contribute to pAEMA’s ability to protect dsRNA from nuclease-mediated degradation, as observed here. In *Spodoptera exigua*, the efficacy of RNAi was enhanced by dsRNA complexed with a cationic polymer, which not only protected dsRNA from midgut nucleases but also improved cellular uptake (Christiaens et al. 2018).

Ingestion represents a potential route of dsRNA uptake. In our study, polymer conjugation produced a modest increase in mortality compared with naked dsRNA. This effect is comparable to that previously reported for lipofectamine-mediated delivery (Castellanos et al. 2019), suggesting that both carriers may similarly improve oral dsRNA delivery. However, high mortality was observed in the treatment with the non-conjugated polymer. This may be attributed to the absence of charge neutralization between the polymer and dsRNA. Without neutralization, the polymer’s positive charges remain exposed and can interact non-specifically with cellular membranes or proteins, contributing to the observed toxicity (Vereecken et al. 2026). In contrast, treatments involving polymer–dsRNA complexes, including dsgfp polyplexes, resulted in lower mortality rates relative to the polymer-alone treatment. These observations support the hypothesis that complex formation mitigates polymer-associated toxicity by masking or neutralizing its cationic charge. Further studies are needed to confirm this mechanism, including assessments of the polymer’s intrinsic toxicity and comparisons with fully characterized, charge-neutralized polymer–dsRNA complexes.

The mortality patterns reported in *N. viridula* are consistent with our results. In that species, feeding insects with naked ds*V-ATPase* A resulted in ~ 40% mortality, which increased to ~ 47% with lipofectamine-complexed dsRNA (Sharma et al. 2021a). Notably, P-labeled dsRNA injected into *N. viridula* is efficiently processed into siRNAs, whereas orally administered dsRNA fails to undergo this conversion, indicating poor translocation across the gut epithelium and limited access to the RNAi machinery (Gurusamy et al. 2020). A similar phenomenon occurs in *E. heros*, which shows strong RNAi responses following microinjection but remains largely refractory to oral delivery. In contrast, *H. halys* displays high susceptibility to orally delivered dsRNA, with mortality exceeding 70% for targets such as *IAP, SNF7* and *PP* when insects ingest beans dipped in dsRNA solutions (Mogilicherla et al. 2018).

With respect to diet intake, insects treated with the dsV-ATPase polyplex consumed less food than those treated with the dsgfp control. Reduced feeding may affect the effective dose of dsRNA ingested and could therefore contribute to the variability observed in the oral RNAi response. This pattern may reflect physiological effects of dsRNA-polyplex formulation at the midgut level or the induction of an antifeedant effect. Further studies are needed to determine whether the observed reduction in feeding is caused by the polymer itself, the dsV-ATPase-induced gene silencing, or a combination of both factors.

At the transcriptional level, exposure to ds*gfp* controls resulted in only modest downregulation of target genes. In contrast, polymer-conjugated dsRNA induced a marked increase in transcript levels relative to the ds*gfp* control, suggesting a stress-related or compensatory physiological response potentially associated with the delivery system. For *V-ATPase*, both naked and polymer-conjugated dsRNA delivered orally caused significant gene silencing with a stronger effect compared to the ds*gfp* control. These results suggest that RNAi-mediated knockdown can occur under both delivery conditions, although the magnitude and temporal dynamics may be influenced by the formulation. Previous studies using lipofectamine reported approximately 35% gene knockdown; however, expression levels tended to recover rapidly over time, in contrast to the sustained silencing observed here (Castellanos et al. 2019). In *H. halys*, no statistically significant differences were detected between dsRNA-treated and control groups, although reductions of up to 30% were reported for the *SNF7* gene, indicating that oral RNAi efficiency may vary widely among hemipteran species (Mogilicherla et al. 2018).

The use of dsRNA–polymer formulations introduce the possibility that observed changes in gene expression may result not only from RNAi-mediated effects but also from physiological responses elicited by the formulation itself. This represents an inherent methodological consideration in RNAi studies relying on in vivo delivery, where both carrier-associated and gene-specific effects may occur simultaneously and may not always be fully distinguishable under experimental conditions. Future studies should further separate RNAi-specific effects from potential carrier-associated responses by increasing the temporal resolution of gene expression analysis, especially at early time points, and by incorporating complementary approaches to assess stress-related gene expression and dsRNA uptake efficiency. This information may provide additional insights into both the performance of the delivery systems and their biological effects in vivo.

Sensitivity to RNAi varies considerable among insect species and is influenced by multiple factors, including dsRNA uptake efficiency, nuclease activity, systemic RNAi capacity, and target gene selection (Cooper et al. 2019; Silver et al. 2021; Ahmad et al. 2025). Among insect orders, coleopterans generally exhibit high susceptibility to both injected and orally delivered dsRNA, making them one of the most RNAi-responsive groups reported to date (Bolognesi et al. 2012; Levine et al. 2015; Prentice et al. 2017; Bachman et al. 2020). In contrast, RNAi responses in Hemiptera are more variable and often species dependent. For example, oral delivery of dsawd in *Diaphorina citri* induced gene silencing, leading to developmental abnormalities and mortality (Killiny and Kishk 2017), whereas in *N. viridula* and *E. heros*, robust RNAi responses and high mortality rates have been achieved primarily through microinjection rather than oral delivery (Castellanos et al. 2019; Cagliari et al. 2020a, b; Sharma et al. 2021a, b), highlighting the challenges associated with RNAi-based pest control in some hemipteran species.

*Euschistus heros* demonstrates exceptional susceptibility to RNAi when delivered by microinjection, with immature insects reaching complete mortality within just eight days. Silencing via this route produces pronounced phenotypic and molecular effects, with substantial downregulation of target genes and early physiological phenotypes preceding death. In contrast, oral exposures are markedly less effective, revealing the formidable barriers imposed by nucleases and gut defenses. Polymer-conjugated dsRNA modestly improves oral efficacy but remains far below microinjection performance. The results of this study highlight the value of integrating complementary approaches to understand the barriers and limitations associated with oral RNAi in *E. heros*. Future advances should focus on dsRNA stability and delivery but also on minimizing off-target effects and optimized application strategies, such as repeated exposure and the use of multiple polymers, to maximize RNAi efficacy under field conditions. By overcoming delivery barriers, RNAi holds transformative potential to redefine pest management—enabling precise, species-specific interventions that reduce reliance on conventional insecticides and advance sustainable agriculture.

## Supplementary Information

Below is the link to the electronic supplementary material.

ESM 1 (DOCX 930 KB)

## Data Availability

The data supporting the findings of this study are available from the corresponding author upon reasonable request.
