# Supplementary material for: Oral RNAi in Euschistus heros: Evaluation of Gene Silencing Using Cationic Polymer-Stabilized dsRNA
Source: Neotrop Entomol. 2026 Sep 16;55(1):88. doi: 10.1007/s13744-026-01440-4 (PMC13582158; doi:10.1007/s13744-026-01440-4)
Supplement: Supplementary file 1 [file 13744_2026_1440_MOESM1_ESM.docx]

Supplementary information

## Sequences

Sequence 1 - ds*gfp* 569bp

TAATACGACTCACTATAGGGAGATCGTGACCACCCTGACCTACGGCGTGCAGTGCTTCAGCCGCTACCCCGACCACAYGAAGCAGCACGACTTCTTCAAGTCCGCCATGCCCGAAGGCTACGTCCAGGAGCGCACCATCTTCTTCAAGGASGACGGCAACTACAAGACCCGCGCCGAGGTGAAGTTCGAGGGCGACACCCTGGTGAACCGCATCGAGCTGAAGGGCATCGACTTCAAGGAGGACGGCAACATCCTGGGCCACAAGCTGGAGTACAACTACAACAGCCACAACGTCTACATCATGGCCGACAAGCAGAAGAACGGCATCAAGGTGAACTTCAAGATCCGCCACAACATCGAGGACGGCAGCGTGCAGCTCGCCGACCACTACCAGCAGAACACCCCCATCGGCGACGGCCCCGTGCTGCTGCCCGACAACCACTACCTGAGCACCCAGTCCGCCCTGAGCAAGGACCCCAACGAGAAGCGCGATCACATGGTCCTGCTGGAGTTCGTGACCGCCGCCGGCATCACTCTCGGCATGGACGACCTATAGTGAGTCGTATTAA

Sequence 2 - ds*V-ATPase* *E. heros* 502 bp

TAATACGACTCACTATAGGGAGAGAGAGGGAGGGATCTGTTTCAATTGTAGGAGCTGTATCACCTCCTGGTGGTGACTTCTCAGATCCTGTAACATCTGCCACTCTTGGTATTGTTCAAGTGTTCTGGGGATTGGACAAGAAGCTGGCTCAAAGAAAGCACTTTCCGTCCATCAACTGGCTTATTTCTTACAGCAAGTACATGAGAGCTTTAGACGATTTTTATGACAAGAACTTCCCTGAGTTTGTTCCACTTAGGACAAAAGTTAAAGAAATCCTTCAAGAAGAAGAAGATCTTTCAGAAATTGTACAACTGGTAGGTAAAGCCTCCCTTGCTGAATCTGATAAGATCACTCTTGAAATCGCCAAGCTACTCAAGGATGACTTCCTTCAACAAAACAGTTACTCACCTTATGATCGTTTCTGTCCATTCTACAAGACCGTCGGTATGTTGAAAAACATGATTTCCTTCTACGACCTGGCCCCTATAGTGAGTCGTATTAA

Sequence 3 - ds*act* *E. heros* 509 bp (Castellanos et al. 2019)

TAATACGACTCACTATAGGGAGAGATGACCCAGATCATGTTTGAGACCTTCAACACCCCAGCCATGTATGTCGCCATCCAGGCTGTACTCTCCCTCTATGCCTCCGGTCGTACTACCGGTATTGTACTTGACTCAGGAGATGGTGTCTCCCACACCGTACCCATCTATGAAGGTTATGCCCTTCCCCACGCCATCCTCCGTCTGGATCTTGCTGGACGTGACTTGACTGACTATCTTATGAAGATCCTCACCGAGCGTGGTTACAGCTTCACCACCACCGCTGAAAGGGAAATCGTCAGGGACATCAAGGAAAAACTGTGCTATGTCGCCCTGGACTTTGAGCAGGAAATGGCCACCGCCGCTGCCTCCACCTCCCTGGAGAAGTCCTATGAACTTCCCGACGGTCAGGTCATCACCATCGGTAACGAGAGGTTCCGTTGCCCAGAGGCTCTCTTCCAGCCTTCCTTCTTGGGTATGGAATCTTGTTAATACGACTCACTATAGGGAGA

Figure S1 Agarose gel analysis of naked dsRNA and dsRNA–pAEMA polyplexes. Lanes 1–3 naked ds*V-ATPase*, ds*act*, and ds*gfp*, each showing a distinct band at the expected size. Lanes 4–6 contain the corresponding dsRNA–pAEMA polyplexes, where no bands are detected and the material remains in the loading wells, confirming successful polyplex formation and effective charge neutralization by the polymer.

Figure S2 Degradation of 5 µl of dsRNA polyplex: A. ds*V-ATPase* and B. ds*gfp* solution at 200 ng/µl, mixed with 3 µl of saliva, from *E. heros,* and incubated for 72 hours at 25°C.

Figure S3 Intensity profile graph generated by GelAnalyzer showing electrophoresis results from the ex-vivo degradation experiment of naked and dsRNA polyplex in *E. heros* saliva and hemolymph. A) Degradation of 30 µl at 200 ng/µl of naked and polyplex ds*V-ATPase* and ds*gfp* mixed with 3 µl of *E. heros* saliva, with samples taken at 1, 10, 30, 60, 120, and 180 minutes after incubation at 25°C. B) Degradation of 30 µl at 200 ng/µl of naked and polyplex ds*V-ATPase* and ds*gfp* mixed with 3 µl of *E. heros* hemolymph, with samples taken at 1, 10, 30, 60, 120, and 180 minutes after incubation at 25°C. C) Degradation of 30 µl at 200 ng/µl of naked and polyplex ds*V-ATPase* and ds*gfp* mixed with 3 µl of nuclease-free water, with samples taken at 10, 30, 60, 120, and 180 minutes after incubation at 25°C.

Figure S4 Consumption of an artificial diet mixed with dsRNA, naked or polyplexes, or water by third instar *E. heros* after 48 hours. P-value <0.05.
